# HMGA Proteins in Stemness and Differentiation of Embryonic and Adult Stem Cells

**DOI:** 10.3390/ijms21010362

**Published:** 2020-01-06

**Authors:** Silvia Parisi, Silvia Piscitelli, Fabiana Passaro, Tommaso Russo

**Affiliations:** Department of Molecular Medicine and Medical Biotechnology, University of Naples Federico II, Via S. Pansini 5, 80131 Naples, Italyfabiana.passaro@unina.it (F.P.); tommaso.russo@unina.it (T.R.)

**Keywords:** high mobility group proteins, embryonic stem cells, adult stem cells, gene regulation, regulation of translation, cell reprogramming, LIN28, miRNAs

## Abstract

HMGA1 and HMGA2 are chromatin architectural proteins that do not have transcriptional activity per se, but are able to modify chromatin structure by interacting with the transcriptional machinery and thus negatively or positively regulate the transcription of several genes. They have been extensively studied in cancer where they are often found to be overexpressed but their functions under physiologic conditions have still not been completely addressed. *Hmga1* and *Hmga2* are expressed during the early stages of mouse development, whereas they are not detectable in most adult tissues. *Hmga* overexpression or knockout studies in mouse have pointed to a key function in the development of the embryo and of various tissues. HMGA proteins are expressed in embryonic stem cells and in some adult stem cells and numerous experimental data have indicated that they play a fundamental role in the maintenance of stemness and in the regulation of differentiation. In this review, we discuss available experimental data on HMGA1 and HMGA2 functions in governing embryonic and adult stem cell fate. Moreover, based on the available evidence, we will aim to outline how HMGA expression is regulated in different contexts and how these two proteins contribute to the regulation of gene expression and chromatin architecture in stem cells.

## 1. Introduction

High mobility group A (HMGA) proteins are non-histone chromatin proteins. Their classification as a high mobility group refers to their rapid electrophoretic migration, in part due to their small sizes (10–15 kDa). The analysis of available nucleotide sequences shows that *HMGA1* and *HMGA2* orthologues are present in mammals, birds, fishes and reptiles. *HMGA1* orthologues have been found in 254 species, while 231 organisms have a *HMGA2* ortholog (NCBI Gene data bank). Similar proteins are also present in many taxa, including plants, where it is difficult to assign the homology to HMGA1 or HMGA2, due to the high similarity of these two proteins, which probably evolved from a single ancestor. HMGA1 and HMGA2 are small proteins with a very similar structure that includes three conserved domains, also known as AT-hooks, and a C-terminal domain rich in acidic residues. Each AT-hook domain contains a characteristic stretch of 9 residues, mostly Arg and Lys, which interacts with a stretch of 15 AT bp with high affinity [1,2]. However, depending on the sequence of the DNA cognate site, one molecule of HMGA2 can use only one or two AT-hooks to leave the other/s free to interact in trans with other segments of DNA, thus generating a higher order structure of chromatin. HMGA proteins in solution have little secondary structure, but, when co-incubated with synthetic DNA, they bind to DNA and thus adapt their structure to that of the minor groove of the molecule [3]. Splicing variants have been described encoding HMGA1 and HMGA2 proteins with differences that could be responsible for specific DNA binding properties and/or interaction with partners [4,5].

HMGA proteins are highly expressed during embryonic development as demonstrated by Northern blot and in situ hybridization analyses on mouse embryos, showing that *Hmga1* is expressed until 16.5 dpc (days post coitum), whereas *Hmga2* disappears already at 14.5 dpc [6,7]. *Hmga1* expression was detectable in the yolk sacs of wild-type mice at 9.5 dpc. Its expression decreases at 14.5 dpc and increases in the fetal liver [8]. The available data of whole mount hybridization from MGI (mouse genome informatics) localize *Hmga2* in the somite, tail and eye at 9.5 dpc with a localization confined only in the eye at 13.5 dpc (MGI database). In 14.5 dpc mouse embryos, *Hmga1* is highly expressed in several tissues such as the epidermis, stomach, midgut and hindgut, testis, lung, pancreas, thyroid and thymus primordium (in situ analysis from the MGI database). In later phases of fetal development and in most adult tissues, these two proteins are not detectable [6,7]. Only *Hmga2* was found in preadipocytic proliferating cells, spermatids, and spermatocytes [1].

On the other hand, HMGA proteins are often expressed at very high levels in cancers [1]. These include benign tumors, like for example lipomas, breast fibroadenomas, salivary gland adenomas, hamartomas, and pituitary adenomas, where in most cases, chromosome rearrangements involving the *HMGA2* gene were found [1]. The latter resulted in the expression of truncated forms of the protein or in the fusion of the N-terminus of HMGA2 with the C-terminus of other proteins, always leading to the deletion of the normal 3′ untranslated region of the mRNA. Similar observations were also made concerning *HMGA1* gene rearrangements in lipomas, uterine leiomyomas, pulmonary chondroid hamartomas [9,10]. The causal role of *Hmga2* in the tumorigenesis was supported by the spontaneous development of lipomas and of other types of benign tumors in mice overexpressing *Hmga2* or its truncated forms [11,12,13].

In malignant cancers, elevated levels of either *HMGA1* or *HMGA2* have been frequently found [1]. Their overexpression is in most cases associated with the aggressiveness of the disease, metastatic diffusion and poor survival [1,14,15]. Similar observations were made in hematological malignancies, for both *HMGA1* and *HMGA2*. Despite the large number of studies focusing on HMGA proteins, their functions in normal cells are not definitively understood. The main hurdle in addressing this issue is probably that most of the experimental data that could contribute to the understanding of HMGA proteins was obtained in tumor cells. Indeed, in many cases, what is observed in cancer cells does not necessarily take place, exactly in the same fashion, in a normal cell. For example, in the case of HMGA proteins, it was demonstrated that they accumulate in senescent cells and that their knockdown by RNAi resulted in a partial bypass of the senescence induced by oncogenic Ras [16]. These data seem to conflict with the above-mentioned observation that support a role of HMGA proteins in neoplastic transformation, but actually indicate that the HMGA functions are context-dependent, thus they act as effective oncogenes, probably only when senescence-inducing mechanisms are turned off.

We find that the multiple roles of HMGA proteins in physiological contexts are intriguing and still not completely understood. Thus, considering that HMGA proteins in cancer are already extensively described and reviewed [1,17,18], this review is focused on the function of HMGA proteins in non-tumor contexts. HMGA proteins are expressed, in normal conditions, mostly during embryonic development, in embryonic stem cells (ESCs) and in adult stem cell populations. ESCs and adult stem cells are able to self-renew and to differentiate in vitro, giving rise to specific cell types, thus mimicking the events that take place in vivo during early developmental stages (ESCs) or the events that regulate tissue homeostasis (adult stem cells). These characteristics make these cells a powerful tool to study in vitro molecular mechanisms underlying self-renewal and differentiation, but they also represent a potential source of specific cell types for drug screenings and cell-replacement therapy. Considering that a huge effort is still required to understand stem cell biology and that many reports indicated important roles of HMGA proteins in stem cell contexts we believe that this review can help reconstitute the complex puzzle representing the molecular mechanisms governing stem cell fate. Thus, here we review the findings addressing HMGA functions in regulating stem cell behavior and outline the complex regulation of HMGA expression and how these proteins contribute to chromatin dynamics.

## 2. The Phenotypes of *Hmga* KO and Transgenic Mice

The phenotypes observed in *Hmga1* and *Hmga2* knockout mice clearly indicate their crucial role in the different phases of mouse development. *Hmga1* KO mice show a complex phenotype including the downregulation of insulin receptors and consequent glucose intolerance and cardiac hypertrophy [19,20]. In these KO mice, the globin switch with fetal globin (βH1 and ζ) fails, thus remaining higher in 9.5- and 14.5-dpc *Hmga1*−/− yolk sacs and in 14.5-dpc fetal livers compared with wild-type tissues [8]. This suggests a role of *Hmga1* in regulating hematopoiesis, in agreement with data obtained in ESCs (see below). *Hmga2* gene KO in mice is responsible for a specific phenotype, also known as pigmy, consisting in reduced weight at birth, shortened head, and a body weight of adult mice that is about 40% of the normal weight [6]. Further than the adipose tissue, the growth of skeletal muscles is also severely affected in *Hmga2* KO mice [21]. *Hmga1* and *Hmga2* probably partially compensate for the absence of each other, as suggested by the dramatic phenotype of the *Hmga1/Hmga2* double KO mice, which show a 70% reduction of the body weight at birth, which is not compatible with survival [22]. Interestingly, *Hmga1/Hmga2*-null mice also showed embryonic lethality, indicating that the HMGA proteins play a critical role during embryonic and/or fetal development [22].

Mice overexpressing *Hmga2* show a characteristic phenotype, including overgrowth with an enlargement of the adipose tissue [11]. This phenotype is consistent with the *Hmga2* roles in humans, where its chromosomal rearrangement, leading to mRNA truncation, was described in a patient showing dramatic somatic overgrowth [23].

## 3. HMGA Functions in Embryonic Stem Cell (ESC) Stemness and Differentiation

In physiological contexts, *Hmga1* is highly expressed in mouse ESCs (mESCs) and decreases during differentiation, playing a crucial role in their lymphohematopoietic differentiation. Indeed, *Hmga1* KO ESCs showed a decrease in the formation of T-cell precursors balanced by an increase in B cell generation. Moreover, the absence of *Hmga1* expression induces a reduction in monocyte/macrophage population and an increase in megakaryocyte precursor numbers, erythropoiesis, and globin gene expression [8]. *HMGA1* is also expressed in undifferentiated human ESCs (hESCs) and its levels decline during differentiation [24]. This expression profile agrees with the observation that the ectopic expression of *HMGA1* blocks the differentiation of hESCs, maintaining the undifferentiated state, as demonstrated by the resulting high levels of stemness markers such as *OCT4*, *NANOG*, *SOX2* and *MYC*. Accordingly, the suppression of *HMGA1* by RNA interference in hESCs resulted in a downregulation of pluripotency genes, *SOX2*, *OCT4*, c*MYC* and *LIN28*, likely by direct interaction of HMGA1 with their promoters, at least *SOX2*, *MYC* and *LIN28* [24]. In addition to pluripotency regulation, *HMGA1* also shows a role in enhancing the establishment of a pluripotent stem cell phenotype through cell reprogramming. In 2006, Takahashi and Yamanaka demonstrated that somatic cells can be reprogrammed into induced pluripotent stem cells (iPSCs) by four transcription factors (TFs): OCT4, SOX2, KLF4, c-MYC (OSKM), also called the Yamanaka cocktail [25]. These cells, as ESCs do, can self-renew indefinitely and differentiate into any cell types, thus becoming an ethically acceptable alternative to ESCs for their application in in vitro disease models, drug screenings and cell replacement therapies. For these reasons, starting from the Yamanaka discovery, many stemness-related factors were tested to improve reprogramming efficiency [25]. Among them, *HMGA1* led to a two-fold increase in human mesenchymal stem cell (MSC) reprogramming efficiency when added to the Yamanaka cocktail. This effect is due to the HMGA1-dependent expression of a subset of pluripotency- associated genes during the early phases of the reprogramming process [24].

The data reported for *HMGA2* in hESCs are consistent with a role of HMGA proteins in controlling stem cell identity and differentiation through regulation of gene expression in these cells. Indeed, *HMGA2* is expressed at high levels in hESCs and is further up-regulated during a short time window in the very early stages of hESC differentiation [26]. In these cells, HMGA2 may interact with nucleosomes, possibly contributing to a specific state of chromatin domains, which, in turn, may be critical in gene expression regulation, thus governing both self-renewal and differentiation of hESCs. This hypothesis is supported by microarray analysis upon *HMGA2* knockdown showing that HMGA2 regulates the expression of genes linked to self-renewal and proliferation as well as mesodermal differentiation in hESCs [27]. A more recent paper has indicated another role of *HMGA2* in hESCs in addition to that of gene expression regulation. Yu and colleagues demonstrated that HMGA2 is required to protect stalled replication forks against nucleolytic collapse in hESCs as well as cancer cells that, exhibiting fast DNA replication cycles, frequently encounter stalling of replication forks [28].

In mESCs, *Hmga2* is barely detectable but it promptly accumulates upon the induction of differentiation [29,30] into epiblast like cells (EpiLCs) and neuronal precursors to decrease in the late phase of neural differentiation [30]. The high expression of *Hmga2* in EpiLCs is in agreement with the expression in hESCs that actually behave as mouse epiblast stem cells. *Hmga2* induction during the early phases of mESC differentiation is required for the exit from the naïve state. Indeed, the suppression of *Hmga2*, by either gene KO or silencing, blocks the differentiation program. On the other hand, persistence of high levels of *Hmga2* during differentiation perturbs the cell cycle and increases the apoptosis rate of differentiating cells [31], thus indicating that *Hmga2* expression must be tightly controlled to allow the proper ESC differentiation. The mechanisms through which *Hmga2* affects mESC differentiation involve the HMGA2-dependent regulation of differentiation genes. Upon the exit from the undifferentiated state, mESCs acquire a phenotype resembling that of the epiblast stem cells that are primed for further differentiation [32]. This phenomenon is dependent on the activation of a large array of genes under the control of the TF OTX2 [32,33]. ChIP experiments demonstrated that HMGA2 is necessary for the engagement of OTX2 with cognate enhancers whose activation is required for the transcription of genes that control the exit from the undifferentiated state [16]. *Hmga2* itself is one of these genes, thus a feedforward loop based on the induction of *Hmga2* expression sustains the change in the expression profile of differentiating mESCs.

In agreement with the described role of HMGA2 in allowing the differentiation of mESCs [30] and the reduced size of skeletal muscle in *Hmga2* KO mice [21], the overexpression of both the wt and truncated form of *Hmga2* in mESCs specifically favors myogenic differentiation without affecting other cell lineages [29].

As observed in the case of HMGA1, HMGA2 can promote adult cell reprogramming toward a stem cell phenotype. Human dermal fibroblasts or mouse embryonic fibroblasts (MEFs) can be directly converted into induced neural stem cells (iNSCs) by simply transducing them with the transcription factor SOX2 [34]. The co-expression of SOX2 with HMGA2 significantly increases the efficiency of direct reprogramming [35]. Interestingly, hMSCs derived from umbilical cord blood, which expresses higher levels of *HMGA2* compared to dermal fibroblasts, are more prone to be reprogrammed into iNSCs [35]. Thus, higher levels of HMGA2 are correlated with higher reprogramming efficiency. This role of HMGA2 in improving direct reprogramming into stem cells is in agreement with the requirement of HMGA2 in reprogramming mouse embryonic fibroblasts into iPSCs. Indeed, MEFs KO for *Hmga2* showed a strong decrease in reprogramming efficiency, although some reprogrammed colonies can be obtained [30].

## 4. HMGA Proteins in Adult Stem Cells

*Hmga1* is enriched in intestinal stem cells (ISCs). In conditional transgenic mice, *Hmga1* overexpression amplifies Wnt/*β*-catenin signalling to enhance self-renewal and expand the ISC compartment. HMGA1 also helps to “build” an ISC niche by expanding the Paneth cell compartment. Moreover, HMGA1 resulted in the organization of ISCs into three-dimensional organoids in vitro. These experimental data indicated a role for HMGA1 in intestinal homeostasis by maintaining the stem cell pool and fostering terminal differentiation to establish an epithelial stem cell niche [36,37].

In adult stem cells, *Hmga2* has been detected in NSCs and progenitor cells of the subventricular zone of newborn mice [38]. The amount of *Hmga2* present in these cells declines with age, becoming undetectable in old mice. Experiments in *Hmga2* KO mice demonstrated that this protein is necessary for the self-renewal of NSCs. In addition, this effect could be based on HMGA2-mediated regulation of gene expression. Indeed, HMGA2 sustains self-renewal of NSCs by downregulating p16^Ink4a^ and p19^arf^, and ChIP experiments have indicated a direct interaction of HMGA2 with the *JunB* gene, which in turn regulates p16^Ink4a^ and p19^arf^ [38]. It is worth noting that in NSCs, *Hmga1* does not decrease with age, thus appearing not to be involved in this regulatory mechanism. However, these data are not completely in agreement with the observations of Kishi and co-workers [39] who, through in utero electroporation experiments, demonstrated an involvement of both HMGA proteins in conferring the neurogenic potential to neural precursor cells (NPCs) in vivo. In this experimental setting, the silencing and overexpression of *Hmga1* and/or *Hmga2* affect chromatin condensation in NPCs of mouse neocortex, and the decline of *Hmga* expression parallels the increasing condensation of the chromatin. In agreement with ChIP-seq data upon overexpression of *Hmga* in mESCs [40], these data seem to confirm that *Hmga* ectopic expression has a more general effect on chromatin structure, instead of an effect targeted to specific gene loci.

The role of *Hmga2* in neurogenesis is also supported by further evidence. *Hmga2* is expressed at a high level in the neocortex at E12.5, but rapidly declines, becoming undetectable at E15.5. The overexpression of *Hmga2* causes a rearrangement of the neocortical layers, with the *Hmga2* overexpressing cells retained in the profound layers, while the in utero silencing of *Hmga2* has an opposite effect, shifting the *Hmga2* knockdown cells towards more superficial layers [41]. Moreover, in *Hmga2* KO mice, the number of cells present in the gut enteric nervous system that form neurospheres at E14.5 is similar to the number found in the wild type gut, but this number significantly declines at P0 and in the adult mice. In all cases, the size of the neurospheres is always reduced in the *Hmga2* KO mice [38].

In the fetal hematopoietic compartment, HMGA2 is necessary to sustain the self-renewal capacity of mouse hematopoietic stem cells (HSCs). Ikeda and coworkers first observed that high levels of *Hmga2* are associated with an expansion of the hematopoietic compartment, including HSCs [42]. When HSCs from the fetal liver of *Hmga2* KO mouse embryos were transplanted in irradiated congenic mice, the frequency of donor-derived HSCs is reduced compared to that observed when wild type cells were transplanted, thus indicating a decreased self-renewal capacity of *Hmga2* KO HSCs [43]. The mechanism underlying the HMGA2 activity in these cells was not explored in detail, but the expression profile analysis of *Hmga2* KO HSCs suggested that the *Igf2bp2* gene could be directly regulated by HMGA2 and, at least in part, responsible for the observed phenotype. The reduced size of skeletal muscle found in *Hmga2* KO mice (see above) supports the idea that HMGA2 can directly affect *Igf2bp2* gene expression by regulating its transcription. Indeed, *Igf2bp2* mRNA and protein are decreased in *Hmga2* KO myoblasts and, on the contrary, *Hmga2* overexpression in these cells results in the accumulation of *Igf2bp2* mRNA and protein. IGF2BP2 seems to be an important player downstream of HMGA2, as its ectopic expression partially reverts the *Hmga2* KO muscle phenotype [21].

In human HSCs, *HMGA2* was detected for the first time in CD34-positive cells [44], and appears to play a role similar to that described in mouse, as the colony-forming potential of cord blood CD34+ cells is significantly reduced upon its silencing [5].

A detailed analysis of the *HMGA2* contribution to human HSC differentiation showed that the suppression of *HMGA2* leads to a decrease of myeloid progenitor cells but has no effects on the differentiation of these cells. On the contrary, HMGA2 is necessary for both erythroid precursor propagation and differentiation [45]. Of note, Calvazzana-Calvo and colleagues described a crucial role of HMGA2 in the hematopoietic compartment. Indeed, the therapeutic benefit obtained by lentiviral *β*-globin gene transfer in an adult patient with severe *β*E/*β*0-thalassaemia was correlated to the transcriptional activation of *HMGA2* mRNA in erythroid stem/progenitor cells, which is accompanied by a benign cell expansion [46].

The role of HMGA2 in sustaining self-renewal of adult stem cells possibly by blocking their differentiation was also described in hMSCs. Indeed, *HMGA2* overexpression blocks the differentiation of hMSCs into the osteogenic lineage by limiting the expression of the osteogenic factor RUNX2 and, conversely, the down-regulation of endogenous *HMGA2* promotes osteogenic differentiation [47,48,49].

Interestingly, HMGA2 may have a dual role in differentiation of adult stem cells. Indeed, in hMSCs the silencing of *HMGA2* expression, in addition to its effect in promoting osteogenic differentiation, also leads to a severe impairment of adipogenesis [47]. This role can be mediated by the cooperative interaction of HMGA2 with TF STAT3, working as an adipogenic inducing factor [50]. These observations are in agreement with the phenotype of *Hmga2* KO mice that shows a striking reduction in adipose tissue, and with transgenic mice overexpressing *Hmga2* showing somatic overgrowth and, in particular, increased abundance of fat and lipomas [11,23].

All these experimental data point to a general role of HMGA proteins in regulating the balance between self-renewal and differentiation of stem cells (Table 1). This is also supported by the observations obtained in tumor and cancer stem cells. Indeed, *HMGA1* is highly expressed in human colon tumor stem cell lines and its silencing increases stem cell quiescence, reduces self-renewal and sphere-forming efficiency, and recovers the expression of NUMB, an endocytic protein promoting asymmetric division that is typical of normal stem cells [51]. The same effect was observed in human brain tumor stem cells where *HMGA1* is highly expressed and upon its silencing the CD133+/CD15+ stem cell population is reduced [52]. In these cells, HMGA1 negatively regulates *NUMB* both at transcriptional level and post-transcriptionally through the regulation of the RNA binding protein MSI1 and the miR-146a expression [53].

## 5. HMGA Proteins as Regulators of Chromatin Architecture and Gene Expression

Many of the experimental data reported above suggest that HMGA proteins contribute to regulating chromatin structure, and thus influencing gene expression. A direct binding to chromatin of HMGA1 or HMGA2 was described in several different conditions. These proteins preferentially bind to A/T-rich sequences near, or overlapping with, binding sites of TFs orchestrating the assembly of multi-subunit protein–DNA complexes (enhanceosomes) by modifying the chromatin structure in an ATP-independent fashion [54]. One example of this function is the *IFN-β* gene promoter. Upon viral infection, the transcription of the *IFN-β* gene depends on the recruitment of several TFs, including NFkB, to an enhancer element within the *IFN-β* gene promoter. The assembly of this complex is dependent on the interaction of HMGA1 with an A/T-rich sequence present in the promoter [55]. Another example of the role of HMGA in enhanceosome formation is that of the *IL-2Rα* gene. *Hmga1* is upregulated upon stimulation of T cells and binds to A/T-rich sequences in the *IL-2Rα* gene promoter inducing a chromatin remodeling that allows the accessibility of regulatory cis-elements to several TFs, like ELF-1, STAT5 and others [56]. Duncan and colleagues reported that HMGA proteins interact with A/T-rich sequences placed on the surface of, or close to, positioned nucleosomes, which hamper the binding of sequence-specific TFs on *IL-2Rα* and *α-B-crystallin* gene promoters [57]. In the stem cell context, many experimental data have indicated a role of HMGA proteins in allowing the recruitment of TFs at specific chromatin regions to modulate the behavior of these cells. HMGA1 can specifically bind two AT-rich sequences in the GATA-1 upstream activating element and down-regulate GATA-1 promoter activity to allow the proper megakaryocte and erythroid differentiation of mESCs [8]. In hESCs, as mentioned above, HMGA2 may interact with nucleosomes, thus contributing to a specific state of chromatin domains [26,27]. Furthermore, HMGA2 is found associated to OTX2 binding sites (containing A/T repetitions) to assist this TF in the pioneering of new enhancers to allow proper differentiation of mESCs [30].

One of the effects of HMGA seems to be that of removing the nucleosomal constraints that prevent the formation of the TF-DNA complexes. This effect can be explained by the observation that the chromatin binding sites of the HMGA proteins are like those of histone H1 indicating that HMGA proteins compete with H1 for binding to linker DNA, thereby inducing a loosening of the chromatin structure [58,59]. However, the molecular mechanism underlying the replacement of histone H1 by HMGA proteins and the consequent chromatin opening is not well understood. Histone H1 eviction from the chromatin is not enough to allow the TFs to access their binding elements. Nevertheless, HMGA proteins can bind to both nucleosomes and chromatin remodelers indicating a possible role in eviction and/or mobilization of core histones during transcriptional regulation [59]. Other than this global role of HMGA proteins in changing the chromatin state, data obtained upon modulation of HMGA expression showed changes in the gene expression profile, thus indicating a specific role of these proteins on the transcription of a discrete array of putative target genes [5,30,31,60,61]. For example, ChIP experiments demonstrated that HMGA1 binds to the mouse *Brca1* promoter in mESCs, repressing its expression [62]. Microarray analysis of *Hmga1* KO and wt mESCs have identified 87 transcripts increased and 163 decreased in the absence of HMGA1. Many of these target genes showed cell- and tissue-specific regulation by HMGA1 when compared with the results obtained in MEFs, liver, spleen and heart from wt and *Hmga1* KO mice [63]. These differences can be based on the ability of HMGA1 to enhance or suppress the effect of transcriptional activators and repressors by interacting with different partner proteins. However, the mechanisms underlying the binding of HMGA proteins to specific chromatin regions are not understood. While experimental evidence indicated that endogenous HMGA proteins are associated with specific chromatin regions, ectopic expression of either *Hmga1* or *Hmga2* in undifferentiated mESCs results in a diffuse binding of these proteins to chromatin mainly at heterochromatic regions [40]. This discrepancy could be explained by hypothesizing that the strong increase of HMGA proteins by overexpression can also induce a permissive binding to chromatin regions, which in basic conditions, show low binding affinity. Moreover, the concomitant and balanced expression of specific interactors could be necessary to make the binding of HMGA proteins specific.

## 6. Regulation of HMGA Proteins in Different Cellular Contexts

An important advancement in the understanding of HMGA2 functions emerged from the discovery of a complex crosstalk among this protein and other molecules involved in several differentiation programs. Following the discovery of the small RNA lin-4 in 2000, which is able to regulate the translation of the *Lin-14* mRNA through an RNA–RNA interaction [64], the microRNA (miRNA) let-7 was described for the first time in *C. elegans* for its ability to regulate expression of multiple mRNA targets [65]. In mammals, there are several miRNAs belonging to the let-7 family and *Hmga2* mRNA is one of the targets of let-7. Thus, as let-7 induces the downregulation of HMGA2 protein, the expression profile of these two molecules is specular: HMGA2 is expressed in undifferentiated cells, while let-7 miRNAs are expressed in differentiated cells. A further key player in this regulatory mechanism is LIN28. LIN28A and B are two RNA binding proteins that are able to limit the biogenesis of a subset of miRNAs, and in particular those of the let-7 family. The main mechanisms underlying this regulation are based on the direct interaction of LIN28B with the pri-let-7 RNA, preventing the processing of this molecule by the microprocessor complex in the nucleus [64] and of LIN28A with pre-let-7 RNA that prevents Dicer-dependent processing in the cytosol. The latter is mediated by the recruitment of TUT4, which polyuridylates the pre-miRNA [65,66]. Taken together, these data led to unveil the existence of the so called LIN28-let7-HMGA2 axis that dictates the HMGA2 levels. The tight control of this axis regulates many biological processes (some already described above, see References [35,38,43]).

A beautiful example of the effects of the modulation of the LIN28-let7-HMGA2 axis is represented by retinal progenitor cells (RPCs), where let-7 facilitates differentiation. In this context, high levels of *Hmga2* and *Lin28* maintain the stemness of mouse RPCs. During late retinal histogenesis, let-7 increases and negatively regulates the translation of *Hmga2*/*Lin28*. Low levels of *Hmga2*/*Lin28* and high levels of let-7 in RPCs shift the balance from RPC maintenance to their differentiation [67].

The LIN28-let7-HMGA2 axis also fulfills a crucial role in hematopoietic maturation to adulthood in mice. The decrease of *Lin28* in myeloid progenitors parallels the accumulation of mature let-7. The inhibition of let-7 in the adult hematopoietic system recapitulates fetal erythroid-dominant hematopoiesis. Conversely, deletion of *Lin28* or ectopic activation of let-7 in the fetal state induces a shift toward the adult-like myeloerythroid phenotype. Furthermore, the analysis of the effects induced by *Hmga2* ectopic expression indicated that this architectural factor as an effector of LIN28-let-7-controlled myeloerythropoiesis [68].

In human adipose tissue-derived MSCs, let-7 positively regulates osteogenic differentiation by repressing *HMGA2* through direct targeting [47]. Accordingly, induced *LIN28* expression in MSCs reduces the expression of let-7 and up-regulates that of *HMGA2*, which in turn activates the transcription of pluripotency-associated factors, maintaining the stem cell phenotype [69].

Other recent reports have also shown that in hMSCs, the expression of *HMGA2* can be controlled by other miRNAs that might synergize with let-7 in a context-dependent manner [47,48,49]. In MSCs derived from human bone marrow, *HMGA2* repression by the microRNA (miR)-664a-5p is required for proper osteogenic differentiation. Consistently, in these cells, overexpression of *HMGA2* counteracts the stimulatory effect of miR-664a-5p on osteogenic differentiation [49]. Moreover, Gao and colleagues showed that during osteogenic differentiation of MSCs, the direct targeting of miR-98 to *HMGA2* mRNA is required to properly fulfill the differentiation program [48]. As said before, IGF2BP2 is one of the downstream targets of HMGA2; in addition, it has also a critical role in its regulation. IGF2BP2 is an RNA binding protein that regulates the translation of many mRNAs [21,70]. Among these mRNAs are *HMGA2* itself and *LIN28* mRNAs whose translation is favored by IGF2BP2 [71,72,73]. This intricate network of reciprocal regulation is further complicated by the observation in tumor cells that RPSAP52, a non-coding RNA (ncRNA) transcribed from a ribosomal protein pseudogene, has an apparently important role in the network. Indeed, the pseudogene encoding RPSAP52 overlaps with the *HMGA2* gene. At the C/G skew present in the *HMGA2* gene promoter, the ncRNA forms an R-loop with the genomic DNA, thus favoring chromatin decompaction and the transcription of the *HMGA2* gene [73]. Thus, RPSAP52 induces the accumulation of HMGA2 through the transcriptional activation of the cognate gene. However, RPSAP52 has also a regulatory role in the cytoplasm, where it binds to IGF2BP2 [72]. The interaction of RPSAP52 with *IGF2BP2* promotes the binding of IGF2BP2 to the *HMGA2* mRNA, thus favoring its translation. It is worth noting that RPSAP52 does not affect the binding of IGF2BP2 to other known mRNA targets, like *HMGA1*, *NRAS* and *IGF1R*. RPSAP52 may also act through an additional competing endogenous RNA (ceRNA)-based mechanism, as it can titrate several miRNAs targeting *HMGA1* and *HMGA2*, thus leading to their increased expression [74]. These observations are made in tumor cells, and thus, it would be interesting to understand if HMGA2 can undergo the same positive regulation by the transcribed pseudogene RPSAP52, at both transcriptional and post-transcriptional levels.

In mESCs, although many miRNAs have a fundamental role in controlling gene expression, targeting specific mRNAs and regulating the exit from the pluripotent state [75,76,77,78,79,80], the let-7 miRNA family does not act in this crucial phase of differentiation. Indeed, in undifferentiated ESCs and EpiLCs, let-7 genes are poorly transcribed and their processing to an active form is blocked by LIN28 proteins [31,65,78,79]. Here, beyond the self-induction of *Hmga2* expression through the cooperation with OTX2, *Hmga2* expression is regulated by LIN28 in a let-7-independent manner [31]. Indeed, LIN28 binds a highly conserved element in the 3′ UTR of *Hmga2* mRNA, and this negatively controls its translation. This mechanism prevents the inappropriate accumulation of HMGA2 during differentiation that would modify the proliferation and physiological apoptosis of ESCs. On the other hand, upon the exit of mESCs from the undifferentiated state and the transition into epiblast-like cell states, LIN28 accumulates along with HMGA2. This induction of *Lin28* is dependent on the direct interaction of OTX2 and HMGA2 with *Lin28* genes [31].

**Figure 1:**
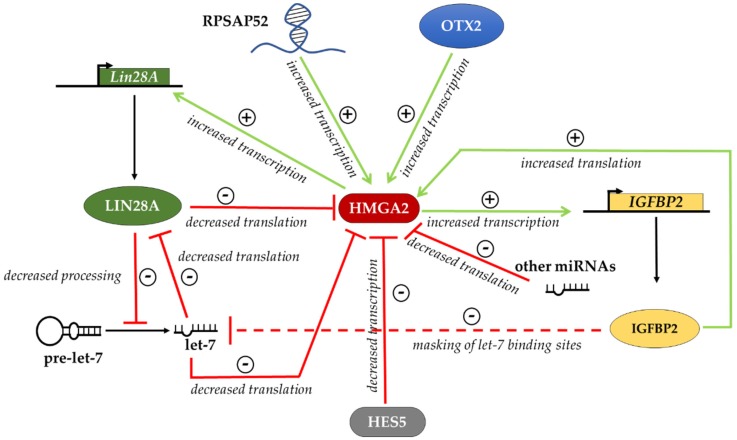
Regulatory network controlling HMGA2 protein levels. HMGA2 levels are regulated by RNA binding proteins, like IGF2BP2 and LIN28, which, through the interaction with the *HMGA2* mRNA, increase or decrease the translation, respectively. HMGA2 expression is also downregulated by HES5 at the transcriptional level and by let-7 and other miRNAs at the post-transcriptional level. The sign + indicates improved expression of the target, the sign—indicates impaired expression of the target.

Several reports [21,30,31,47,48,49,70,71,73] mentioned above allow us to outline at least in part the complex regulation of HMGA2 expression at various levels, which we have summarized in Figure 1. HMGA2 levels are positively regulated by the ncRNA RPSAP52, which activates the transcription of the *HMGA2* gene and, together with IGF2BP2, activates the translation of the *HMGA2* mRNA. HMGA2 levels are negatively regulated by let-7 miRNAs. In addition, IGF2BP2 could regulate let-7 levels by masking miRNA target sites on various mRNAs, thus favoring let-7 degradation. This is expected to result in a further increase of HMGA2 biosynthesis. In addition, both HMGA2 and IGF2BP2, through independent mechanisms, favor the expression of LIN28 and this in turn contributes to the downregulation of let-7 miRNAs. Therefore, there are several positive feedback loops, all contributing to increase the amount of HMGA2. Two questions remain to be addressed. First, in cells expressing HMGA2, LIN28 and IGF2BP2, a negative regulator is necessary to balance the positive loops described above. In mESCs differentiating into EpiLCs, where let-7 miRNAs do not accumulate, this indispensable negative regulator of *Hmga2* could be LIN28, which hampers the translation of *Hmga2* mRNA. Second, in differentiating adult stem cells, the exit from the “undifferentiated” state could be triggered by the inhibition of *Lin28* expression that results in accumulation of let-7 miRNAs. Furthermore, other factors can contribute to downregulation of HMGA2 levels in cell-specific contexts, such as other specific miRNAs possibly synergizing with let-7 [47,48,49], as well as specific transcriptional repressors such as HES5 [81] or the same HMGA1 [22].

**Figure 2:**
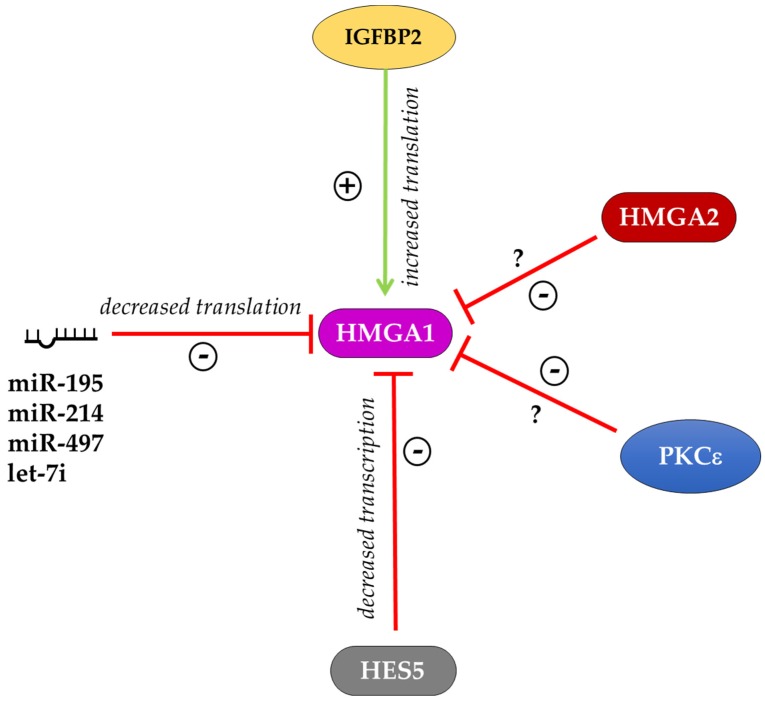
Schematic representation of HMGA1 expression regulation. HMGA1 is positively regulated by IGFBP2 at the post-transcriptional level. The transcriptional repressor HES5 and several miRNAs downregulate the expression of HMGA1. The kinase PKCε and HMGA2 repress HMGA1 expression through mechanisms that are still unknown. The sign + indicates improved expression of the target, the sign—indicates impaired expression of the target.

On the other side, little experimental evidence is available on the regulation of HMGA1 expression. In the mouse myoblast cell line, C2C12, a PKCε–HMGA1 signaling axis was described that regulates skeletal muscle differentiation. In these cells, the kinase PKCε down-regulates *Hmga1* expression, which in turn leads to the increased expression of myogenic differentiation genes to allow myotube formation [82]. The work of Bansod and colleagues reported that the transcriptional repressor HES5 regulates the timing of neurogenesis and gliogenesis, controlling the expression of epigenetic factors such as HMGA1 and HMGA2. Indeed, the overexpression of *Hes5* in mice inhibited neuronal differentiation from NSCs, while gliogenesis was also accelerated and enhanced. In these mice, *Hmga1* and *Hmga2* expression was suppressed in the neocortical regions. By contrast, in the *Hes5* knockout (KO) mice, the transition of neurogenesis and gliogenesis was delayed and this effect was accompanied by the upregulation of *Hmga1* and *Hmga2*. In utero electroporation of shRNA targeting *Hmga1* and *Hmga2* in *Hes5* KO mice showed that the transition timing of layer-specific neurogenesis and astrogenesis was regulated at least in part by the expression levels of *Hmga1* and *Hmga2* genes [81]. Interestingly, the data obtained in *Hmga1* and *Hmga2* KO MEFs suggested the existence of a feedback loop among these two factors that reciprocally regulates each other. Indeed, in *Hmga1* KO MEFs, the expression of HMGA2 protein is increased and in *Hmga2* KO MEFs the expression of HMGA1 protein is increased [22]. This effect could be mediated by post-transcriptional regulation (for example through miRNAs) considering that the mRNA levels of *Hmga1* and *Hmga2* did not show significant differences in KO MEF lines [22]. A post-transcriptional regulation of *Hmga1* expression through specific miRNAs is well described in different cell contexts [83,84,85]. In human cervical and colorectal cancer cells, the expression of *HMGA1* is high and is inversely correlated with miR-214 expression. Luciferase assays and western blot demonstrated that miR-214 overexpression reduces the level of *HMGA1* and counteracts its effects on proliferation, migration and invasion in cervical and colorectal cells [83]. During myogenic differentiation of C2C12 myoblasts, *Hmga1* expression is inversely correlated with the expression of miR-195/497. The abundance of the HMGA1 protein was reduced in C2C12 cells after the induction of the myogenic program, which is opposite to the upregulated expression of the miR-195/497. The *Hmga1* 3’ UTR luciferase reporter assays in C2C12 cells showed that the ectopic miR-195/497 significantly repressed the luciferase activity. Consistent with this data, ectopic expression of miR-195/497 reduced the level of HMGA1 in C2C12 cells [84]. In human bladder cancer cell lines, let-7i is downregulated compared to normal cells, whereas *HMGA1* is highly expressed. In these cells, the transfection of let-7i mimics reduced *HMGA1* mRNA and protein expression suggesting that *HMGA1* is a target for let-7i [85]. All these factors negatively regulate HMGA1 expression in both humans and mice (Figure 2). To our knowledge, only the RNA binding protein IGFBP2 positively regulates HMGA1 expression at the translational level by binding the *HMGA1* mRNA 3′UTR and inhibiting its degradation [86]. Thus, further investigation is required to understand the signaling and the transcriptional regulators that contribute to control HMGA1 expression.

## 7. Conclusions

The data discussed in this review point to a crucial role of HMGA proteins in the control of the balance between self-renewal and differentiation in embryonic and adult stem cells (Table 1). These data suggest that HMGA1 fulfills the same functions in human and mouse ESCs: its high expression in undifferentiated cells from both organisms is required for self-renewal maintenance and its downregulation is necessary for proper differentiation [8,24]. In adult stem cells, further experimental evidence is required to outline the function of HMGA1, not only as a determinant of self-renewal [36], but also in influencing specific stem cell differentiation programs. In the case of HMGA2, the picture is more complicated. *HMGA2* is highly expressed in hESCs [24,28], whereas in the mouse counterpart, it is barely detectable [29,30], and the effects due to its modulation are different. This can be explained by considering that mouse ESCs behave differently from human ESCs that actually resemble mouse EpiLCs in which *Hmga2* starts to be expressed [30] and where its function needs to be better described. In adult stem cells, where HMGA2 is highly expressed in both humans and mice, this protein seems to be required to allow self-renewal and to block differentiation [5,38,42,43,45,47,48,49]. However, the decline of HMGA2 during the differentiation of adult stem cells has to be tightly controlled to allow efficient differentiation [5,45,47,48,49]. All these effects are possibly due to the ability of HMGA proteins to change the epigenetic landscape by modulating chromatin organization.

**Figure 3:**
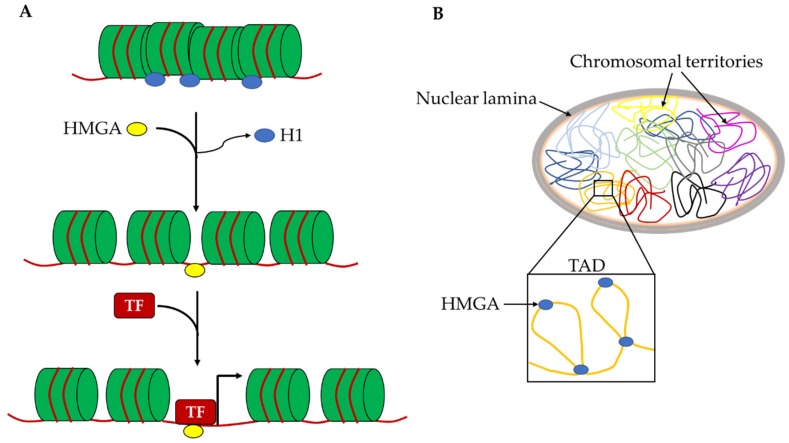
Schematic representation of hypothetical roles of HMGA proteins in the chromatin architecture. (**A**) Hmga proteins can allow the accessibility to pioneer TFs on naïve chromatin by displacing H1 histone. (**B**) Hmga2 proteins can contribute to the organization of chromosomal territories and their subdomains (TADs) by generating specific DNA loops. H1: histone H1; TF: transcription factor; TAD: topological associated domain.

For this reason, the HMGA effects on cell reprogramming are very intriguing. HMGA2 is required to efficiently obtain mouse iPSCs and the overexpression of both *Hmga1* and *Hmga2* leads to an increase in reprogramming efficiency. Cell reprogramming mechanisms are mostly based on the epigenetic remodeling of the starting cells to allow the profound changes in chromatin organization and gene expression needed to acquire the reprogrammed phenotype. Most factors that are able to improve reprogramming efficiency, called reprogramming enhancers, are epigenetic modulators. In this context, HMGA proteins can be considered reprogramming enhancers and their role in this mechanism is possibly correlated with their ability to change chromatin organization at different levels. Indeed, the binding affinity of HMGA proteins for A/T rich sequences allows one to recognize countless points on the genome and thus intragenic or intergenic regulatory elements (enhancers and silencers), as well as gene empty regions. In this way, HMGA proteins may have a double role in changing the epigenetic profile. At the local level, they allow the accessibility to pioneer TFs on naïve chromatin to allow the entry of canonical TFs, probably by displacing H1 histone and removing chromatin constrains that block the assembly of TF-DNA complexes (Figure 3A). At the global nuclear level, these proteins, as architectural factors, can generate specific DNA loops that contribute to the organization of chromosomal territories and their subdomains such as topological associated domains and lamina associated domains (Figure 3B). Both these activities of HMGA proteins give a crucial contribution to the regulation of gene expression.

## Figures and Tables

**Table 1:** Phenotype resulting from alteration of HMGA expression in stem cells and in cell reprogramming. Abbreviations: embryonic stem cells (ESCs), mesenchymal stem cells (MSCs), intestinal stem cells (ISCs), neural stem cells (NSCs), hematopoietic stem cells (HSCs).

HMG	Organism	Cell Type	HMG Level	Phenotype	References
HMGA1	mouse	ESCs	KO	Impairment of lymphohematopoietic differentiation	[8]
HMGA1	human	ESCs	overexpression	Block of differentiation, maintenance of the undifferentiated phenotype	[24]
HMGA1	human	ESCs	silencing	Downregulation of pluripotency genes	[24]
Hmga1	human	MSCs	overexpression	Improvement of reprogramming into iPSCs from MSCs	[24]
HMGA1	mouse	ISCs	overexpression	Enhancement of self-renewal	[36]
HMGA2	human	ESCs	silencing	Decrease of self-renewal and mesodermal genes and increase endodermal genes	[27]
HMGA2	human	ESCs	silencing	Increase in the amount of fragmented DNA	[28]
HMGA2	mouse	ESCs	overexpression	Improvement of myogenic differentiation	[29]
HMGA2	mouse	ESCs	Silencing or KO	Block of differentiation, maintenance of the undifferentiated phenotype	[30]
HMGA2	mouse	ESCs	overexpression	Impairment of cell cycle and apoptosis during differentiation	[31]
HMGA2	mouse	Embryonic fibroblasts	KO	Sever impairment of reprogramming into iPSCs	[30]
HMGA2	human	Dermal fibroblasts	overexpression	Improvement of reprogramming into induced neural stem cells	[34]
HMGA2	mouse	Embryonic fibroblasts	overexpression	Improvement of reprogramming into induced neural stem cells	[34]
HMGA2	mouse	NSCs	KO	Decrease of self-renewal	[38]
HMGA2	mouse	HSCs	overexpression	Increase of self-renewal	[42]
HMGA2	mouse	HSCs	KO	Decrease of self-renewal	[43]
HMGA2	human	HSCs	silencing	Decrease of self-renewal, differentiation impairment	[5,45]
HMGA2	human	MSCs	overexpression	Block of differentiation	[47,48,49]
HMGA2	human	MSCs	silencing	Improvement of osteogenic differentiation	[47]
